# Smartphone and application use in self-management of chronic kidney disease: a cross-sectional feasibility study

**DOI:** 10.1590/1516-3180.2022.0078.R2.09082022

**Published:** 2022-10-03

**Authors:** Christielle Lidianne Alencar Marinho, Orlando Vieira Gomes, Geraldo Bezerra da Silva, Paulo Adriano Schwingel

**Affiliations:** I MSc. Nurse and Doctoral Student, Health Sciences Program, Universidade de Pernambuco (UPE), Recife (PE), Brazil.; II MD, MSc. Nephrologist and Assistant Professor, School of Medicine, Universidade Federal do Vale do São Francisco (UNIVASF), Petrolina (PE), Brazil.; III MD, PhD. Physician and Professor, Department of Internal Medicine, School of Medicine, Universidade Federal do Ceará (UFC), and School of Medicine, Public Health and Medical Sciences Graduate Programs, Universidade de Fortaleza (UNIFOR), Fortaleza (CE), Brazil.; Universidade de Fortaleza, School of Medicine, Fortaleza, CE, Brazil; IV PhD. Sports Physiologist and Associate Professor, Human Performance Research Laboratory, Universidade de Pernambuco (UPE), Petrolina (PE), Brazil.

**Keywords:** Renal insufficiency, chronic, Treatment adherence and compliance, Smartphone, Self-management, Dialysis, Kidney disease, Health technology, Telehealth, Health monitoring, Digital health

## Abstract

**INTRODUCTION:**

Smartphone and application use can improve communication and monitoring of chronic diseases, including chronic kidney disease, through self-management and increased adherence to treatment.

**OBJECTIVE:**

To assess smartphone use in patients with chronic kidney disease on dialysis and their willingness to use mobile applications as a disease self-management strategy.

**DESIGN AND SETTING:**

This was a cross-sectional study of chronic kidney disease patients on hemodialysis in the São Francisco Valley in the Northeast Region, Brazil.

**METHODS:**

The questionnaire developed by the authors was administered between April and June 2021. Cronbach's alpha coefficient for the construct was 0.69. Associations between the dependent and independent variables were determined using univariate analysis. Multivariate analysis with logistic regression analysis was also performed.

**RESULTS:**

A total of 381 patients were included, of whom 64% had a smartphone, although only 3.1% knew of a kidney disease-related application. However, 59.3% believed that using an application could help them manage their disease. Having a smartphone was associated with treatment adherence, higher educational attainment, and higher per capita income. Educational attainment remained an independent factor in multivariate analysis.

**CONCLUSION:**

More than 64% of patients had a smartphone, although few knew of applications developed for kidney disease. More than half of the population believed that technology use could benefit chronic kidney disease treatment. Smartphone ownership was more common among the younger population, with higher educational attainment and income, and was associated with greater adherence to hemodialysis sessions.

## INTRODUCTION

Chronic kidney disease (CKD) is a worldwide public health problem, with approximately 10% of the world population having some degree of CKD. It has significant social and financial implications for both developed and developing countries.^1–3^

International estimates further indicate that the number of people who need renal replacement therapy (RRT) will increase from 2,618 million in 2010 to 5,439 million by 2030.^4^ However, not everyone who needs RRT can access the treatment, because it is not universally covered worldwide.^5^ In Brazil, RRT is universally covered by the Unified Health System. According to the Brazilian Society of Nephrology, the estimated number of new patients undergoing dialysis in 2019 was 45,852 – a 7.7% increase from 2018, along with a 3.9% mean increase in CKD prevalence in the same period.^6^

Adherence to treatment poses an immense challenge for patients with CKD, their relatives, and health teams. The importance of individualized care has been emphasized, including realistic patient-centered goals and shared decision-making between the health team and patient. For this strategy to be effective, the patient's cognitive function, health knowledge, socioeconomic factors, and treatment experiences must be considered.^7,8^

Hence, a viable alternative is to align therapeutic strategies with effervescent technological growth and include this as a tool to achieve better health outcomes via mobile health (mHealth). According to the International Telecommunication Union, 66.6% of the world's population were using mobile Internet at the beginning of 2021. The number of smartphones in use has increased by 7% per year, with an average of more than one million new smartphones coming into use every day.^9^

Even though health technology is used in high-income countries, the widespread use and accessibility of mobile phones have enabled its proliferation in low- and medium-income countries, thereby reaching more people in limited-resource settings.^10^ Recent studies show that mobile devices have improved regular communication and monitoring between health professionals and their patients, as well as adherence to medication use and lifestyle changes.^11–13^

The coronavirus disease 2019 (COVID-19) pandemic has caused rapid unprecedented growth in the use of technology in the health field. However, barriers and challenges–such as patients' lack of knowledge and Internet connectivity, health professionals' limited competence in mHealth, and financial challenges–can hinder the adoption of such interventions.^14^

Thus, to obtain optimal results with this tool, it is important to know the target population of the technology, understand the current limitations, and assess the individuals' knowledge of this resource and willingness to use it.

## OBJECTIVE

The objective of this study was to assess the use of smartphones by CKD patients on dialysis and their willingness to use mobile applications as a strategy for disease self-management.

## METHODS

This was an analytical cross-sectional quantitative study of CKD patients on hemodialysis at a renal treatment reference service in the São Francisco Valley, in the Northeast Region of Brazil.

The eligibility criteria were as follows: age ≥ 18 years and undergoing treatment for > 3 months. Individuals who reported cognitive deficits in their medical records or self-reported disabilities that prevented them from answering the research questions were excluded. A total of 443 patients were registered at the dialysis center, 401 of whom were eligible to participate in the study. Twenty individuals did not agree to participate; therefore, the sample included 381 subjects.

Data were collected using a questionnaire developed by the authors regarding sex, age, marital status, religion, skin color/race, educational attainment, per capita income, hemodialysis time in treatment, kidney disease etiology, associated diseases, use of smartphones, use of applications, use and knowledge of applications for CKD, use of additional tools to cope with and manage the disease, and non-attendance at dialysis sessions in the previous month (https://doi.org/10.6084/m9.figshare.20051600). The instrument to assess the use of mobile technologies in the treatment of CKD was evaluated three times. Research on reliability and reproducibility involved 10 patients, aged 40 to 75 years, undergoing hemodialysis. The instrument items were assessed using Cronbach's alpha for internal consistency. The instrument's reliability was measured by calculating the agreement and estimating kappa coefficients. The Cronbach's alpha coefficient for the construct was 0.687, demonstrating moderate reliability in the three small-group assessments. The supplementary material available at https://doi.org/10.6084/m9.figshare.20051600 analyzes the individual questions and demonstrates maximum agreement values (1.00) for 10 of the 15 questions. In addition, all questions in the instrument demonstrated very high reliability, with values of > 0.90.

Data were collected between April and June 2021 via interviews conducted by trained researchers. Interviews were conducted in a dialysis room while the patients were undergoing treatment. On the day of the interview, a trained researcher conducted a structured face-to-face interview using a standardized questionnaire (SM1) with suitable space for each patient. The patients were asked direct questions and the responses were classified by the interviewer according to the alternatives in the questionnaire.

The answers were typed and stored in regular Excel spreadsheets (Microsoft Corporation, Redmond, Washington, United States, Release 12.0.6662, 2012) and exported to the SPSS computer program (SPSS Inc., Chicago, Illinois, United States, Release 16.0.2, 2008). Descriptive statistical analysis was performed with categorical variables presented as absolute and relative frequencies. Continuous variables were reported as mean ± standard deviation (SD) after data normality was determined using the Kolmogorov-Smirnov test. For inferential analysis, continuous data were analyzed using the Student's t-test for independent samples or one-way analysis of variance. Age and mobile phone use were correlated using Pearson's correlation coefficient. In the univariate analysis, the association between the dependent variable (having a smartphone) and each independent variable (sex, marital status, age group, religion, skin color/race, educational attainment, income, time in treatment, and non-attendance to dialysis) was calculated using Pearson's chi-square test or Fisher's exact test. Variables with P ≤ 0.20 in these analyses were selected for multivariate analysis with logistic regression, performed with the stepwise technique. Unadjusted and adjusted odds ratios (OR) and 95% confidence intervals (95% CI) were calculated. Statistical analyses were two-tailed, and statistical significance was set at P < 0.05.

The Research Ethics Committee of the Amaury de Medeiros Integrated Health Center (CISAM, in Portuguese) approved this research on May 20, 2020, under register number 4.044.382 (CAAE:31246220.1.0000.5191). The participants were informed of the study objective and the procedures they would undergo. Participants then signed an informed consent form agreeing to their voluntary participation in the research.

## RESULTS

The patients' ages ranged from 19 to 92 years, with a mean age (± SD) of 50.8 (± 16.0) years. Most participants were male (n = 240; 63.0%), had completed middle school (n = 129; 33.3%), and earned an income ranging from one to two times the minimum wage (n = 286; 75.1%). The minimum wage at the time was R$ 1,100.00 (US$ 202.00). The sample characteristics are listed in Table 1.

**Table 1:** Sample characterization. São Francisco Valley, Brazil, 2021 (n = 381)

Variables		n	%	95% CI
**Sex**				
	Female	141	37.0%	32.2–42.1
	Male	240	63.0%	58.0–67.7
**Age**				
	≤ 44 years	143	37.5%	32.8–42.5
	45–64 years	157	41.2%	36.4–46.2
	65–74 years	48	12.6%	9.6–16.3
	≥ 75 years	33	8.7%	6.2–11.9
**Marital status**				
	Single	102	26.8%	22.4–31.5
	Married	206	54.1%	48.9–59.2
	Divorced	39	10.2%	7.4–13.7
	Widow(er)	34	8.9%	6.3–12.3
**Religion**				
	Catholic	244	64.0%	59.0–68.9
	Evangelical	110	28.9%	24.4–33.7
	Others	13	3.4%	1.8–5.8
	No religion	14	3.7%	2.0–6.1
**Race/skin color**				
	Multiracial	256	67.2%	62.2–71.9
	Black	53	13.9%	10.6–17.8
	White	72	18.9%	15.1–23.2
**Educational attainment**				
	Illiterate, or elementary school not completed	82	21.5%	17.5–26.0
	Elementary school completed and/or middle school not completed	129	33.9%	29.1–38.9
	Middle school completed and/or high school not completed	58	15.2%	11.8–19.2
	High school completed and/or higher education not completed	94	24.7%	20.4–29.3
	Higher education completed	18	4.7%	2.8–7.4
**Per capita income**				
	Less than 1 time the minimum wage (< US$ 200.00)	58	15.2%	11.8–19.2
	From 1 to 2 times the minimum wage (from U$ 200.00 to 400.00)	286	75.1%	70.4–79.3
	From 3 to 5 times the minimum wage (> U$ 400.00 to 1,000.00)	25	6.6%	4.3–9.5
	More than 5 times the minimum wage (> U$ 1,000.00)	12	3.1%	1.6–5.4
**Time in treatment**				
	Less than 1 year	77	20.2%	16.2–24.60
	From 1 to 2 years	88	23.1%	19.0–27.7
	From 3 to 5 years	101	26.5%	22.1–31.2
	From 5 to 10 years	69	18.1%	14.4–22.4
	More than 10 years	46	12.1%	9.0–15.8
**Non-attendance to treatment sessions in the previous month**				
	Yes	72	18.9%	15.1–23.2
	No	309	81.1%	76.8–84.9

CI = confidence interval.

Although more than 64% of participants had smartphones, only 12 (3.1%) knew about kidney disease-related applications (Table 2). The proportion of kidney patients on hemodialysis who used additional treatment strategies was 14.4% (95% CI: 11.1–18.4). However, approximately 60% of the patients considered that using a mobile application could help manage kidney disease.

**Table 2:** Use and knowledge of mobile phones. São Francisco Valley, Brazil, 2021 (n = 381)

Variables		n	%	95% CI
**Have a smartphone**				
	Yes	245	64.3%	59.3–69.1
	No	136	35.7%	30.1–40.7
**Applications installed**				
	Yes	209	54.9%	49.7–59.9
	No	25	6.6%	4.3–9.5
	Do not know what an application is	11	2.9%	1.5–5.1
	Do not have a smartphone	136	35.7%	30.9–40.7
**Knows of kidney disease-related applications**				
	Yes	12	3.1%	1.6–5.4
	No	369	96.9%	94.6–98.4
**Believes that mobile applications may help manage the kidney disease**				
	Yes	226	59.3%	54.2–64.3
	No	55	14.4%	11.1–18.4
	Do not know	100	26.2%	21.9–31.0
**Uses treatment strategies other than dialysis**				
	Yes	55	14.4%	11.1–18.4
	No	326	85.6%	81.6–88.9
**Additional strategies used**				
	Physical activity	18	4.7%	2.8–7.4
	Physical therapy	7	1.8%	0.7–3.8
	Nutritional therapy	4	1.0%	0.3–2.7
	Psychological therapy	15	3.9%	2.2–6.4
	Religion/Spirituality	4	1.0%	0.3–2.7
	Various therapies	7	1.8%	0.7–3.8
	Does not use additional strategies	326	85.6%	81.6–88.9

CI = confidence interval.

Having a smartphone was associated with adherence to treatment, higher educational attainment, and higher per capita income (Table 3). The mean age of the patients who had a smartphone (44.7 ± 13.5 years) was statistically lower (P < 0.001) than that of the patients who did not have one (61.7 ± 14.2 years).

**Table 3:** Relationship between sociodemographic characteristics, clinical variables, and mobile phone use. São Francisco Valley, Brazil, 2021 (n = 381)

Variables		Has a smartphone				P
		Yes (n = 245)		No (n = 136)		
		n	%	n	%	
**Sex**						
	Female	95	38.8%	46	33.8%	0.337
	Male	150	61.2%	90	66.2%	
**Marital status**						
	Single	74	30.2%	28	20.6%	< 0.001
	Married	138	56.3%	68	50.0%	
	Divorced	22	9.0%	17	12.5%	
	Widowed	11	4.5%	23	16.9%	
**Religion**						
	Catholic	150	61.2%	94	69.1%	0.104
	Evangelical	73	29.8%	37	27.2%	
	Others	9	3.7%	4	2.9%	
	No religion	13	5.3%	1	0.7%	
**Race/skin color**						
	Multiracial	163	66.5%	93	68.4%	0.896
	Black	34	13.9%	19	14.0%	
	White	48	19.6%	24	17.6%	
**Educational attainment**						
	Illiterate, or elementary school not completed	24	9.8%	58	42.6%	< 0.001
	Elementary school completed and/or middle school not completed	77	31.4%	52	38.2%	
	Middle school completed and/or high school not completed	43	17.6%	15	11.0%	
	High school completed and/or higher education not completed	84	34.3%	10	7.4%	
	Higher education completed	17	6.9%	1	0.7%	
**Per capita income**						
	Less than 1 time the minimum wage (< US$ 200.00)	40	16.3%	18	13.2%	0.043
	From 1 to 2 times the minimum wage (from U$ 200.00 to 400.00)	174	71.0%	112	82.4%	
	From 3 to 5 times the minimum wage (> U$ 400.00 to 1,000.00)	21	8.6%	4	2.9%	
	More than 5 times the minimum wage (> U$ 1,000.00)	10	4.1%	2	1.5%	
**Time in treatment**						
	Less than 1 year	48	19.6%	29	21.3%	0.830
	From 1 to 2 years	60	24.5%	28	20.6%	
	From 3 to 5 years	67	27.3%	34	25.0%	
	From 5 to 10 years	42	17.1%	27	19.9%	
	More than 10 years	28	11.4%	18	13.2%	
**Non-attendance to treatment sessions in the previous month**						
	Yes	37	15.1%	35	25.7%	0.011
	No	208	84.9%	101	74.3%	

Moreover, according to the OR calculated for the association of baseline characteristics with mobile phone use, only educational attainment remained an independent factor in smartphone acquisition (Table 4). In addition, the other clinical variables analyzed were not related to mobile phone use nor to kidney disease-related mobile applications.

**Table 4:** Odds ratios of the association between baseline characteristics and mobile phone use. São Francisco Valley, Brazil, 2021 (n = 381)

Variables		Smartphone use					Odds ratio	
		Yes (n = 245)		No (n = 136)		Crude (95% CI)	Adjusted (95% CI)
		n	%	n	%		
**Marital status**							
	Single, divorced or widowed	107	43.7%	68	50.0%	1.00	1.00
	Married	138	56.3%	68	50.0%	1.38 (0.88–2.17)	1.29 (0.85–1.96)
**Educational attainment**							
	Illiterate to middle school completed	144	58.8%	125	91.9%	1.00	1.00
	High school to higher education completed	101	41.2%	11	8.1%	7.97 (4.09–15.52)	7.70 (3.80–15.01)
**Per capita income**							
	2 times the minimum wage or less (≤ U$ 400.00)	214	87.3%	130	95.6%	1.00	1.00
	More than 2 times the minimum wage (> U$ 400.00)	31	12.7%	6	4.4%	1.00 (0.36–2.80)	1.08 (0.39–3.00)
**Non-attendance to treatment sessions in the previous month**							
	Yes	37	15.1%	35	25.7%	1.00	1.00
	No	208	84.9%	101	74.3%	0.65 (0.37–1.12)	0.64 (0.36–1.11)

CI = confidence interval.

## DISCUSSION

Few studies have assessed the use of innovative technologies, including smartphones and applications, as auxiliary methods for treating CKD patients to increase their treatment adherence. Low adherence to CKD treatment has been associated with a greater probability of disease progression and higher mortality.^15^ The study participants were predominantly male, multiracial, married, catholic, with low educational attainment and low income. This reflects the epidemiological profile of the Brazilian population on dialysis. Approximately 65% of the studied patients had a smartphone, and more than half of them used applications in their daily routine. The most used applications were social media, such as WhatsApp, Facebook, and Instagram. Few participants knew of an application to help with kidney treatment. However, more than half of the participants still considered it important and believed it could help them to manage their health conditions. Moreover, smartphone use was associated with income, educational attainment, and adherence to hemodialysis treatment.

A global study investigated CKD epidemiology in 2017 and found a higher prevalence of women in the initial stages of CKD, whereas there were more men in the final stages; moreover, the mortality rates were higher among men.^16^ This may be explained by the harmful effects of testosterone combined with unhealthy lifestyles among men, accelerating their decline in kidney function.

The mean age of the study population was 50.8 years, corroborating other studies conducted in Brazil, wherein the most prevalent age range was from 50 to 60 years.^17^ In a study conducted in Iceland, the mean age of patients with terminal CKD was 63 years, while in another study of 1,174 individuals from Sri Lanka, the mean age was 58.7 years.^18,19^

Studies on CKD conducted both within Brazil and in other countries found similar economic profiles and educational attainments to the present study population. Socially disadvantaged people worldwide face a disproportionate kidney disease burden.^2,6,20,21^ It is important to understand the educational and economic situation of patients who are receiving care to provide them with effective treatment.

Recent technological advancements, combined with the COVID-19 pandemic, have led more people to embrace the alternatives offered by virtual media. Hence, technology that was exclusive to developed countries and economically advantaged people has become accessible and desired by a larger significant portion of the population.^22^

A study of 949 patients on dialysis in the United States showed that 81% of them had smartphones, 72% reported using the Internet, and 60% were interested in using mHealth to manage their health.^23^ Another study conducted on patients on dialysis in Australia found that 83.5% of them had mobile phones, although only 36.6% used applications.^24^ In the present study, this percentage was smaller, which points to the lower purchasing power of patients on dialysis in Brazil. Nevertheless, despite not knowing about any CKD applications, the patients believed that CKD applications could be effective.

One barrier to the implementation of this technology is the limited knowledge of the potential benefits of CKD applications for both users and health professionals. While health professionals recognize the potential of CKD applications, they lack the knowledge, time, and skill to search, assess, and recommend reliable applications, thus highlighting that these technologies need support policies and better publicization.^25^

Health teams must be trained to both use and encourage the use of applications, as they are agents who promote health education, and whom patients trust.^10^ There are Portuguese applications aimed at CKD patients; for example, Renal Health, which has multiple tools such as a smart medication box with reminder alarms, monthly examination charts, liquid and diet control, and general information on kidney disease.^26^

Age, marital status, educational attainment, and income were associated with smartphone use. Younger, single people with higher educational attainment and income tend to have smartphones, in contrast to older, married individuals with lower educational attainment and income. These results corroborate those of other studies in which age, educational attainment, and income were factors associated with smartphone use.^23,27,28^

A primary objective of introducing mobile phone use to promote health self-management is to increase treatment adherence. Patients with CKD must adhere to four treatment pillars: hemodialysis, restricted fluid intake, diet, and medication use. Regarding hemodialysis, only 18.9% of the participants in this study were non-adherent to therapy. Smartphone use was associated with treatment adherence. Thus, it can be inferred that mobile phone use is an interesting tool for increasing adherence. Despite not using specific CKD applications, participants belonged to instant message groups that exchanged information on the disease, its treatment, difficulties, and challenges (data not shown). These platforms allow them to share their afflictions and experiences, generating empathy and consequently energy to continue the treatment.^29^ A systematic review demonstrated that 70% of the studies reported statistical associations between social support and adherence to treatment; moreover, other studies identified social and family support as protective factors against non-adherence to treatment.^30^

Generally, adhering to a given treatment is similar to acquiring a new habit in which information is obtained and incorporated into the routine. However, understanding the person's perceptions and difficulties and becoming acquainted and establishing ties with them simplifies this process.^31^

The possibility of introducing mobile technology into the routine of patients with CKD is very promising, as it can potentially add knowledge and empowerment to their treatment. The patients were interested in this possibility; therefore, health services that treat them should encourage application use and provide the necessary information to promote the technology, including monthly examination results, limits of the liquid they can drink, diet, and medication prescriptions.

Thus, it is important to identify individuals with greater difficulties and barriers to technological access. This will help in allowing mHealth interventions to equitably reach as many people as possible. Application developers must consider the needs of both older adults and those with low literacy to diminish the digital gap between users and non-users. Hence, campaigns to enable older adults to use mobile technologies and increase their health literacy may help to reduce the inequalities caused by technological progress.^32^

Few studies have addressed CKD patients and their interest in and use of smartphones to help promote health among these individuals in Brazil, which makes this research relevant as a bridge to efficiently implementing such resources in the country. A limitation of this study is the single-period and single-service data collection. Thus, although the associations between the variables were assessed, causality between them was not.

## CONCLUSION

More than 64% of CKD patients on dialysis treatment had a smartphone, and 54.9% used applications. Although few patients knew of applications aimed at kidney disease, more than half of them believed that such technology use may benefit CKD treatment. Having a smartphone was more frequent among younger patients with higher educational attainment and income and was also associated with greater adherence to hemodialysis sessions.
